# Calyceal Rupture Secondary to Nephrolithiasis: A Case Report Emphasizing Early Diagnosis and Management

**DOI:** 10.7759/cureus.68305

**Published:** 2024-08-31

**Authors:** Gabriel Cruz, Daniel T Jones, Maria T Lugue, Manvir Heer, Christopher Pace, Linsey Bui, Scott A Silver

**Affiliations:** 1 Internal Medicine, Touro University Nevada, Henderson, USA; 2 Internal Medicine, Touro University California, Vallejo, USA; 3 Internal Medicine, Kansas City University, Joplin, USA; 4 Internal Medicine, Valley Hospital Medical Center, Las Vegas, USA

**Keywords:** percutaneous nephrolithotomy, ureteroscopy, renal colic, perinephric urinoma, hydronephrosis, ureteral obstruction, nephrolithiasis, calyceal rupture

## Abstract

Calyceal rupture, defined as the extravasation of urine from the renal calyces into the perinephric or paranephric spaces, typically results from increased intrapelvic pressure due to urinary tract obstruction. This condition can lead to the formation of a perinephric urinoma and severe complications, such as infection, abscess formation, and impaired renal function. Timely diagnosis and management are crucial to prevent these adverse outcomes. Calyceal rupture often results from urolithiasis, with other causes including strictures, tumors, and congenital abnormalities. The rupture occurs when intrapelvic pressure exceeds the tensile strength of the calyceal walls, leading to urine leakage and potential inflammation or sepsis. Calyceal ruptures are quite rare, with their exact incidence not well-documented due to the infrequency of the condition and potential underreporting. Although relatively uncommon, the condition is more prevalent in individuals with recurrent nephrolithiasis and other predisposing factors. Timely recognition and intervention, guided by imaging studies such as non-contrast CT scans, are essential. Conservative management with medical therapy is effective in many cases, but surgical intervention may be necessary for larger stones or complications. This report presents the case of a 36-year-old female with calyceal rupture secondary to nephrolithiasis, presenting with severe flank pain. Upon initial presentation, the patient underwent a thorough workup, including imaging studies, appropriate medical management, and continuous monitoring. She was stabilized, her pain was effectively managed, and she was discharged with a scheduled outpatient follow-up. This case highlights the importance of early diagnosis, comprehensive management, and vigilant monitoring in preventing complications and promoting favorable outcomes.

## Introduction

Calyceal rupture is defined as the extravasation of urine from the renal calyces into the perinephric or paranephric spaces, typically due to increased intrapelvic pressure resulting from an obstruction in the urinary tract. This condition can lead to the formation of a perinephric urinoma, which is a collection of leaked urine around the kidney. The significance of calyceal rupture lies in its potential to cause severe complications, including infection, abscess formation, and impaired renal function. Prompt diagnosis and treatment are crucial to prevent these adverse outcomes and preserve renal function [1].

Calyceal rupture usually results from an acute increase in intrapelvic pressure due to an obstruction in the urinary tract. The most common cause is urolithiasis, where a stone blocks the flow of urine, causing retrograde flow into the renal pelvis and calyces. Other potential causes include acquired strictures from surgery, infections or trauma, tumors such as renal cell carcinoma, and congenital abnormalities such as posterior urethral valves [2]. When the pressure exceeds the tensile strength of the calyceal walls, a rupture occurs, allowing urine to escape into the surrounding tissues. The obstruction leads to hydronephrosis, a condition characterized by the dilation of the renal pelvis and calyces. If the obstruction persists, the continued accumulation of urine increases the intrapelvic pressure, eventually causing a tear in the calyceal wall. The extravasated urine can lead to perinephric inflammation and, if infected, can result in severe complications such as abscess formation and sepsis [1].

Calyceal rupture is relatively uncommon, with its exact prevalence being difficult to ascertain due to the variable presentation and the fact that it is often underreported. It occurs more frequently in individuals with underlying conditions that predispose them to obstructive uropathy. Nephrolithiasis affects approximately 12% of the population globally, with a higher prevalence in men than women [3]. The lifetime risk of developing kidney stones is estimated to be about 19% in men and 9% in women [4].

Timely diagnosis and management of calyceal rupture are critical to prevent further renal damage and severe complications. Early recognition allows for appropriate intervention to relieve the obstruction, manage symptoms, and prevent infection. Imaging studies, particularly computed tomography scans without contrast (CT w/o contrast), play a crucial role in the early detection of calyceal rupture by revealing both the obstructing pathology and the extent of urine extravasation [2]. Conservative management with medical therapy, such as alpha-blockers like tamsulosin to facilitate stone passage and analgesics for pain control, can be effective in many cases. However, surgical intervention may be required if conservative measures fail or if there are complications like abscess formation or severe infection [5]. Monitoring renal function and ensuring adequate hydration are essential components of the management plan.

## Case presentation

**Figure 1:**
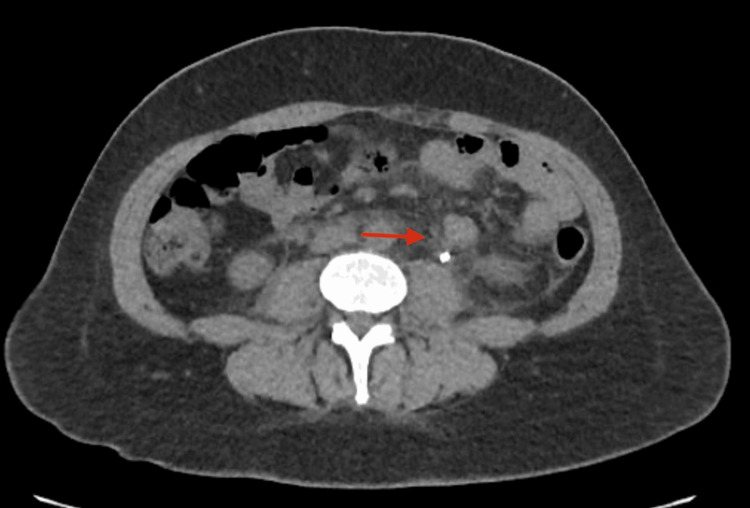
Axial CT abdomen and pelvis without contrast showing trace left perinephric fluid (red arrow) indicative of calyceal rupture

**Figure 2:**
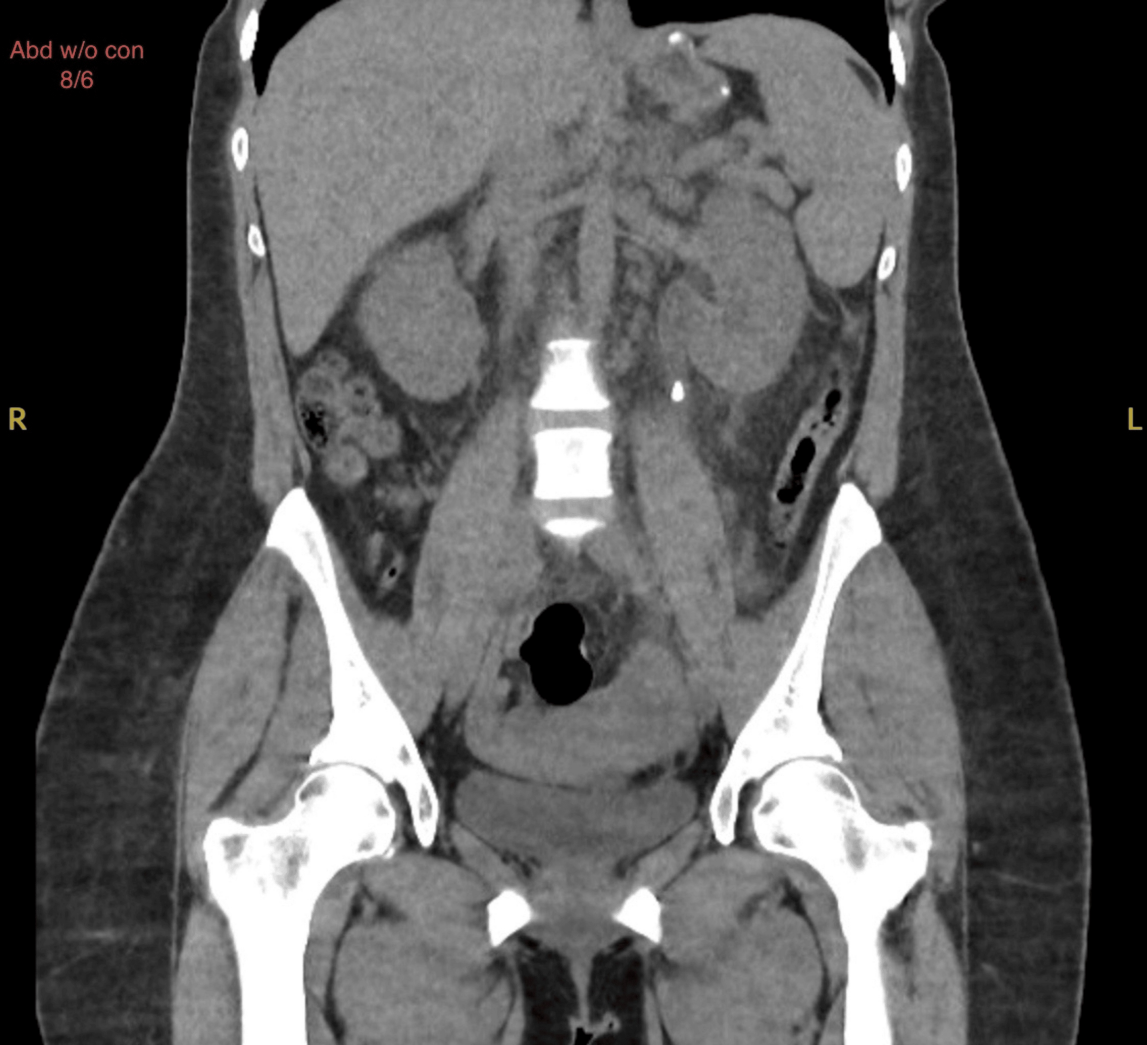
Coronal CT abdomen and pelvis without contrast showing moderate left hydroureteronephrosis (red arrow) and trace left perinephric fluid (yellow arrow) indicative of calyceal rupture The CT scan is a coronal view of the abdomen without contrast (Abd w/o con), dated 8/6. "R" indicates the patient's right side, and "L" indicates the patient's left side.

A 36-year-old female with a medical history of migraines and previous nephrolithiasis presented to the emergency department (ED) with a chief complaint of left flank pain. The pain had started two days prior and was described as constant, sharp, and progressively worsening. In addition, the patient experienced nausea and bile-stained vomiting but denied chest pain, diarrhea, back pain, fever, dysuria, or hematuria. She reported a similar episode approximately 10 years ago related to kidney stones with spontaneous resolution. On initial evaluation, the patient was alert and oriented, with stable vital signs and no apparent distress. Physical examination revealed significant tenderness in the left costovertebral angle upon deep palpation, suggestive of renal colic. The remainder of her physical examination was unremarkable. Diagnostic imaging was performed to evaluate the extent of the condition. A CT scan of the abdomen and pelvis without contrast on day 1 revealed mild left-sided hydronephrosis and a 7 mm stone in the proximal left ureter, along with a 3 mm stone in the left mid to distal ureter. No stones or hydronephrosis were noted in the right kidney. Urology was consulted for potential intervention due to the stone's location and size, and further recommendations were anticipated to guide ongoing management. The lungs and other abdominal organs appeared unremarkable. Follow-up renal ultrasound on day 2 confirmed mild left hydronephrosis without any renal cysts or solid masses, and the right kidney appeared normal. A subsequent CT scan on day 3 demonstrated moderate left hydroureteronephrosis secondary to a 4 mm stone in the left proximal ureter, with trace left perinephric fluid consistent with a calyceal rupture (Figures 1, 2).

The patient was diagnosed with acute symptomatic proximal nephrolithiasis complicated by moderate left hydroureteronephrosis and a calyceal rupture. Renal function tests revealed that the patient's estimated glomerular filtration rate (eGFR) measured at 90>ml/min throughout her stay. The patient's white blood cell count was also within normal range throughout the stay with a peak WBC of 9.63 x 10e3mcL on day 3. Following recommendations from Urology, she was started on appropriate medications and monitored frequently (Tables 1, 2).

**Table 1:** Patient management of ureteral stone passage and symptom control

Treatment component	Medication/action	Dosage/frequency	Additional notes
Stone passage facilitation	Tamsulosin	0.4 mg daily	Administered to facilitate stone passage
Pain control	Morphine	PRN	For severe pain
	Dilaudid (Hydromorphone)	1 mg IV q4h PRN	For severe pain
	Oxycodone-Acetaminophen	10 mg q4h	Significant pain improvement noted
Muscle relaxant	Robaxin (Methocarbamol)	750 mg QID	Hold if drowsiness occurs
Nausea management	Zofran (Ondansetron)	PRN	For nausea
	Reglan (Metoclopramide)	PRN	For nausea
	IV Benadryl (Diphenhydramine)	PRN	For nausea and possible allergic reactions
Fluid management	IV fluids (Isolyte)	125 mL/h	3 doses ordered, continuous infusion
	Fluid I/O monitoring	Daily	Intake and output measured daily
Renal function monitoring	Renal panels	q8h	Renal function closely monitored

**Table 2:** Daily fluid intake and output monitoring

Day of hospital stay	Intake (mL)	Output (mL)
Day 1	1706.98	350
Day 2	440	2400
Day 3	0	700
Day 4	240	Not measured

The patient was admitted to the medical telemetry unit for further management. She remained stable overnight, and her condition was reassessed the following day. The patient reported an overall improvement in her pain and resolution of her nausea. After reviewing the patient's labs and imaging, Urology recommended an outpatient follow-up after four weeks of conservative treatment with tamsulosin.

## Discussion

Calyceal rupture occurs when increased intrapelvic pressure from urinary obstruction exceeds the tensile strength of the calyceal walls, resulting in a tear. This leads to the extravasation of urine into the perinephric or paranephric spaces, potentially forming a perinephric urinoma. The extravasated urine can cause severe inflammation and, if infected, can lead to abscess formation and sepsis [1].

Calyceal ruptures are rare occurrences. The most common cause, accounting for approximately 75% of cases, is obstructive uropathy due to ureteric calculi, where obstructive stones block the flow of urine, resulting in increased pressure and potential rupture [6]. Trauma, whether blunt or penetrating, can directly injure the kidney and induce a rupture. Extrinsic compression from tumors or pregnancy can also lead to obstruction and increased pressure. Intrinsic obstructions such as hematomas or tumors within the kidney or urinary tract can similarly block urine flow and cause rupture [7,8]. In addition, iatrogenic causes, such as complications from medical procedures like lithotripsy or endoscopic interventions, can sometimes result in trauma and subsequent rupture. Severe infections, such as pyelonephritis, can cause inflammation and swelling, leading to increased pressure and potential rupture. Congenital abnormalities, including posterior urethral valves, can cause significant back pressure on the kidneys, ultimately leading to calyceal rupture [9].

Calyceal ruptures can be classified by their location on the kidney, either on the upper pole or lower pole. Lower pole calyceal ruptures are typically caused by less severe obstructions like small kidney stones and present with localized lower flank or abdominal pain, hematuria, and occasionally referred groin pain. These ruptures generally have a favorable prognosis with conservative management, including pain relief, hydration, and antibiotics if infection is present. Diagnosis relies heavily on contrast-enhanced CT scans to assess urinary extravasation. Complications are less common, and surgical intervention is rarely needed unless persistent symptoms or significant complications arise [8,9].

By contrast, upper-pole calyceal ruptures often result from high-impact trauma or significant obstructive uropathy, such as large kidney stones, and present with severe flank pain and hematuria. These ruptures are more prone to complications like perinephric abscesses due to their anatomical location. While conservative management is employed, including pain control and hydration, upper pole ruptures are more likely to require surgical intervention if complications arise [8,10].

Risk factors for nephrolithiasis and subsequent calyceal ruptures include a history of recurrent kidney stones, volume depletion, certain dietary habits, metabolic disorders, and anatomical abnormalities of the urinary tract. Patients with recurrent nephrolithiasis are at a higher risk of developing complications such as hydronephrosis and calyceal rupture due to repeated episodes of obstruction and increased intrapelvic pressure [2].

Patients with calyceal ruptures often present with acute onset of severe flank pain, nausea, and vomiting. These symptoms are similar to those of renal colic or pyelonephritis, creating diagnostic challenges. Physical examination findings are typically nonspecific, making imaging studies crucial for accurate diagnosis. Ultrasound is often the initial imaging technique used due to its availability and non-invasive nature. It can help detect hydronephrosis, perinephric fluid collections, and any evident signs of rupture. However, it is less sensitive than CT in identifying small ruptures and subtle extravasation of urine [11]. Non-contrast CT scans and contrast CT scans are used for diagnosing calyceal rupture, as they can reveal both the obstructing stone and any secondary complications such as hydronephrosis or urine extravasation [3].

Calyceal rupture can range from a manageable condition to a medical emergency, depending on the severity of the obstruction, the extent of urine extravasation, and the presence of complications. It becomes an emergency when there are signs of significant infection, such as fever, chills, or sepsis, which indicate that the extravasated urine may have become infected. Severe pain unresponsive to conventional analgesics, significant hematuria, or evidence of a large urinoma causing pressure on surrounding structures also necessitates urgent intervention. In such cases, immediate imaging and prompt urological consultation are critical to prevent further renal damage and systemic complications. On the other hand, if the rupture is small, the patient's symptoms are mild, and there are no signs of infection or significant hydronephrosis, the condition may be managed conservatively with close monitoring. However, even in less severe cases, timely follow-up and reassessment are essential to ensure resolution and prevent the progression to more serious complications [12].

Treatment of calyceal rupture involves relieving the obstruction, managing pain, and preventing infection. Conservative management includes medical therapy with alpha-blockers like tamsulosin to facilitate stone passage, analgesics for pain control, and antiemetics for nausea and vomiting. This approach is often effective for stones less than 5 millimeters in diameter that are likely to pass spontaneously. Surgical intervention is indicated for larger stones greater than 10 millimeters, failed conservative management, or complications like infection or abscess formation. Specific surgical options include ureteroscopy with laser lithotripsy, percutaneous nephrolithotomy (PCNL), and placement of a nephrostomy tube. Ureteroscopy with laser lithotripsy is a minimally invasive procedure that involves using a ureteroscope to visualize the stone and a laser to fragment it, allowing for easier passage or removal. This method is effective for stones located in the ureter and can be performed with minimal recovery time [3]. PCNL is typically reserved for larger stones or stones located in the kidney that cannot be managed with less invasive techniques. This procedure involves making a small incision in the back and inserting instruments directly into the kidney to remove the stone. It is highly effective for large or complex stones but requires a longer recovery period [3]. In cases where immediate relief of obstruction and drainage of urine is necessary, a nephrostomy tube can be placed percutaneously to bypass the obstruction and allow urine to drain directly from the kidney. This is often a temporary measure used in conjunction with other treatments to manage infection and relieve pressure [3].

Outcomes vary depending on the treatment approach and the underlying cause of obstruction. Conservative management is generally successful for small stones, with most patients experiencing symptom relief and stone passage within weeks. Surgical intervention has higher success rates for larger stones but carries risks of complications such as infection and bleeding. Long-term outcomes are favorable if the obstruction is promptly relieved and any infection is effectively managed. Our case demonstrated a successful outcome with conservative management, highlighting the potential for positive results without the need for invasive procedures [12].

This case underscores the importance of prompt recognition and management of calyceal ruptures. Clinicians should maintain a high index of suspicion in patients with severe flank pain and a history of nephrolithiasis. Early imaging with CT scans are crucial for accurate diagnosis. Treatment should be tailored to the severity of obstruction and the presence of complications, with close monitoring to ensure effective resolution. Future research should focus on optimizing diagnostic protocols and treatment strategies to enhance patient outcomes and prevent recurrence. This could involve studies comparing the efficacy of various imaging modalities and evaluating the long-term outcomes of conservative treatment versus surgery, which would provide valuable insights for clinical decision-making. In addition, this case highlights the need for individualized patient care plans and the importance of being prepared to escalate treatment when conservative measures are insufficient [3].

## Conclusions

This case highlights the critical importance of prompt recognition and management of calyceal rupture due to obstructive uropathy. Calyceal rupture, although uncommon, can lead to severe complications such as infection, abscess formation, and impaired renal function if not diagnosed and treated promptly. Early imaging with CT scans remain the gold standard for accurate diagnosis, providing essential details on the location and size of stones, the extent of hydronephrosis, and the presence of extravasated urine. Conservative management, including medical therapy with alpha-blockers, analgesics, and antiemetics, can be effective for small stones and uncomplicated cases. However, surgical interventions such as ureteroscopy with laser lithotripsy, percutaneous nephrolithotomy, or nephrostomy tube placement are necessary for larger stones, failed conservative management, or complications. The variability in management strategies underscores the need for individualized treatment plans based on the severity of the rupture, the size and location of the stone, and the patient's overall clinical condition. Continuous monitoring and timely follow-up are crucial to ensure resolution and prevent progression to more serious complications. Future research should focus on optimizing diagnostic protocols and treatment strategies to enhance patient outcomes and prevent recurrence. This could involve studies comparing the efficacy of various imaging modalities and evaluating the long-term outcomes of conservative treatment versus surgery, which would provide valuable insights for clinical decision-making. This case also emphasizes the need for clinicians to maintain a high index of suspicion in patients with severe flank pain and a history of nephrolithiasis, ensuring that they are prepared to escalate treatment when conservative measures are insufficient.
